# Exercise for Elderly People

**Published:** 1892-01

**Authors:** Fernand Lagrange


### EXERCISE FOR ELDERLY PEOPLE.

While the elderly man has less capacity for some forms of exercise than the younger adult, he has no less need than the other of the general and local effects of exercise. It is in the earliest period of mature age that the most characteristic manifestations of defects of nutrition—obesity, gout and diabetes, in which lack of exercise plays an important part—are produced; and the treatment of them demands imperiously a stirring up of the vital combustion. Placed between a conviction that exercise is necessary, and a fear of the dangers of exercise, the mature man ought, therefore, to proceed with the strictest method in the application of this powerful modifier of nutrition. It is impossible, however, to trace methodically a single rule for all men of the same age, for all do not offer the same degree of preservation. We might, perhaps, find a general formula for the age at which the muscles and bones have retained all their power of resistance, and at which the heart and vessels begin to lose some of their capacity to perform their functions. The mature man can safely brave all exercises that bring on muscular fatigue, but he must approach with great care those which provoke shortness of breath.—*Fernand Lagrange, M. D.*

